# Is Root Catalase a Bifunctional Catalase-Peroxidase?

**DOI:** 10.3390/antiox6020039

**Published:** 2017-05-25

**Authors:** Vasileia Chioti, George Zervoudakis

**Affiliations:** Technological Educational Institute of Western Greece, Department of Agricultural Technology, Terma Theodoropoulou, Amaliada 27200, Greece; sileia_chioti@yahoo.gr

**Keywords:** aminotriazole, catalase, leaf, peroxidase, root

## Abstract

Plant catalases exhibit spatial and temporal distribution of their activity. Moreover, except from the typical monofunctional catalase, a bifunctional catalase-peroxidase has been reported. The aim of this study was to investigate whether the leaf and root catalases from six different plant species (*Lactuca sativa*, *Cichorium endivia*, *Apium graveolens*, *Petroselinum crispum, Lycopersicon esculentum*, and *Solanum melongena*) correspond to the monofunctional or the bifunctional type based on their sensitivity to the inhibitor 3-amino-1,2,4-triazole (3-AT). The leaf catalases from all species seem to be monofunctional since they are very sensitive to 3-AT. On the other hand, the root enzymes from *Lactuca sativa*, *Cichorium endivia*, *Lycopersicon esculentum*, and *Solanum melongena* seem to be bifunctional catalase-peroxidases, considering that they are relatively insensitive to 3-AT, whereas the catalases from *Apium graveolens* and *Petroselinum crispum* display the same monofunctional characteristics as the leaves’ enzymes. The leaf catalase activity is usually higher (*Lactuca sativa*, *Petroselinum crispum*, and *Solanum melongena*) or similar (*Cichorium endivia* and *Apium graveolens*) to the root one, except for the enzyme from *Lycopersicon esculentum*, while in all plant species the leaf protein concentration is significantly higher than the root protein concentration. These results suggest that there are differences between leaf and root catalases—differences that may correspond to their physiological role.

## 1. Introduction

A wide range of biotic and abiotic environmental factors induce the generation of Reactive Oxygen Species (ROS) and disturb the redox environment of plant cells leading to oxidative stress [1,2]. Hydrogen peroxide (H_2_O_2_) is a very common ROS involved in plant responses, and catalase (CAT) is one of the most important ROS-scavenging enzymes of plants considering that, due to the action of catalases and peroxidases, which decompose this substance, the lifetime of H_2_O_2_ in living tissues is not very long (<1 s) [2,3].

Catalase was the first discovered and characterized antioxidant enzyme [2] and displays spatial and temporal distribution in plant tissues [4]. The “true” (monofunctional) catalases catalyze a dismutation reaction in which an initial H_2_O_2_ molecule is reduced to H_2_O and a second H_2_O_2_ is then oxidized to O_2_ [5]. A second type of the enzyme, mainly found in prokaryotes and some fungi, consists of bifunctional catalase-peroxidases that are structurally distinct proteins and can be distinguished from the monofunctional ones by their relative insensitivity to the inhibitor 3-AT [2]. The bifunctional enzymes, through their peroxidatic activity, can convert H_2_O_2_ to H_2_O with the help of a reducing substrate [5].

According to a recent study from our laboratory, a quite different enzyme activity profile against 3-AT was shown between the leaf and root CAT from basil (*Ocimum basilicum* L.) corresponding to the monofunctional and the bifunctional type, respectively [6]. On the other hand, the bifunctional catalases in the literature to date have been reported to be leaf or shoot enzymes [7,8,9]. In the present study, we examined the leaf and root CAT activity from different plant species and its sensitivity against 3-AT in order to investigate whether the leaf and root catalases correspond to different types of enzyme.

## 2. Materials and Methods

### 2.1. Plant Material

The following plant species were used for this study: *Lactuca sativa* and *Cichorium endivia* (Asteraceae), *Apium graveolens* and *Petroselinum crispum* (Apiaceae), and *Lycopersicon esculentum* and *Solanum melongena* (Solanaceae). Young seedlings were obtained from a nursery and transplanted (after washing the roots in order to remove the soil) in an experimental glasshouse of the Technological Educational Institute of Western Greece, located in Amaliada. Plants were grown aeroponically as previously described using the high level nitrogen nutrition [6], under natural temperature and daylight conditions.

All measurements were conducted 6–7 weeks after transplanting for *Lactuca sativa*, *Cichorium endivia*, *Apium graveolens*, and *Petroselinum crispum* and 8–9 weeks for *Lycopersicon esculentum* and *Solanum melongena*. The leaf samples were taken from healthy, completely expanded leaves. The samples were whole leafs without the midrib (*Apium graveolens*, *Petroselinum crispum*, *Lycopersicon esculentum*, and *Solanum melongena*) or peripheral parts of the leaf (*Lactuca sativa* and *Cichorium endivia*). The root samples were taken from lateral roots. The samples were collected from the plants, wrapped in plastic bags and transferred immediately to the lab for CAT activity and protein concentration estimation. The samples were washed gently with deionized H_2_O and blotted with paper.

### 2.2. Catalase Activity and Protein Concentration

The samples were ground at 4 °C in a porcelain mortar with homogenization buffer containing 100 mM K_2_HPO_4_, pH 7.0, 1 mM EDTA, 0.5 mM phenylmethylsulfonyl fluoride (PMSF), and 0.3% ethanol. The homogenization was carried out using 5 mL of buffer per g fresh sample weight. The homogenate was centrifuged at 3300× *g* for 5 min at 4 °C. The resulting supernatant was subsequently used for both CAT activity and protein concentration estimation. Catalase was assayed by its depletion of H_2_O_2_ [10].

The standard CAT assay consists of mixing (in a 30 °C water bath) 0.85 mL of sample (proper dilution of the homogenate supernatant), 0.05 mL of the homogenization buffer, and 0.1 mL of 0.09 M H_2_O_2_ stock solution (made fresh in homogenization buffer). The linear absorbance decrease during the assay mixture was measured at 253 nm in a Shimadzu UV-1601 spectrophotometer. The absorbance decrease rate of consumed H_2_O_2_ was converted to CAT units (U) from a pure CAT standard curve.

The 3-AT inhibition assay test of CAT consists of mixing 0.85 mL of sample, 0.05 mL of 0.4 M 3-AT (made fresh in homogenization buffer), and 0.1 mL of 0.09 M H_2_O_2_ stock solution. The H_2_O_2_ absorbance decrease of both standard and inhibition assay mixture was measured at 253 nm, in place of the usual wavelength of 240 nm, because at this wavelength the 3-AT spectrum does not interfere significantly with the H_2_O_2_ spectrum (data not shown).

The protein concentration was determined as has been previously described [6].

All reagents were purchased from Sigma (St. Louis, MO, USA) and Merck (Darmstadt, Germany).

### 2.3. Data Analysis

The results were obtained from 3 to 4 independent experiments per plant species and expressed as mean ± standard error of mean (SEM). Each independent experiment was conducted on a different plant and each sample was measured in triplicate. All data were plotted using Microsoft Office Excel 2007. Statistical differences between the means of the plant tissues (leaves and roots) were calculated by implementing a Student’s *t*-test in MS-Excel for statistical level of significance *a* = 0.05.

## 3. Results and Discussion

Catalase, primarily observed and characterized in tobacco leaf, is one of the major systems for the enzymatic removal of H_2_O_2_ in plants [6]. Angiosperm species studied to date (including both monocots and dicots) all contain three catalase genes. The CAT genes’ expression in different tissues of the plant (photosynthetic, vascular, reproductive, and seed) has been classified based on the naming of the tobacco genes [2,11].

**Figure 1:**
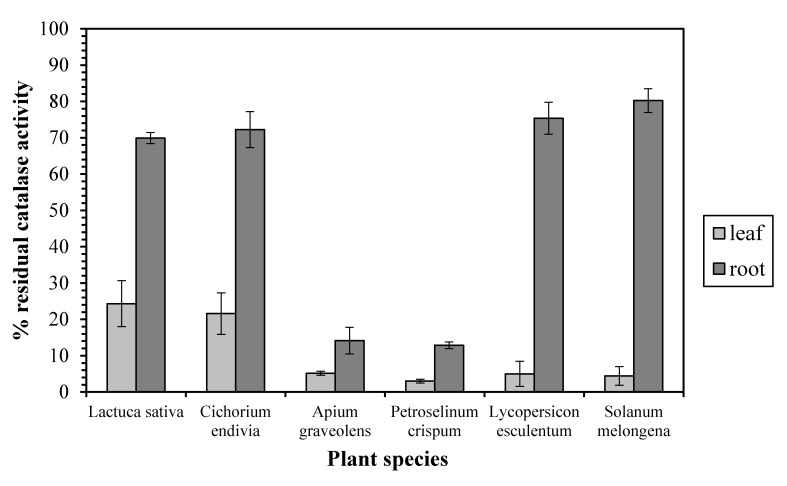
The inhibition effect of aminotriazole on leaf and root catalase activity. Vertical bars represent mean ± SEM (*n* ≥ 3).

Our results exhibit a quite different sensitivity profile between the leaf and root enzyme against the CAT inhibitor 3-AT. In particular, a CAT inhibition assay test in the presence of 20 mM 3-AT revealed that the leaf enzyme’s activity was dramatically decreased (by 75–97%) in all the examined plant species (Figure 1), implying that leaf CAT is monofunctional enzyme. On the other hand, the root CAT from *Lactuca sativa*, *Cichorium endivia*, *Lycopersicon esculentum*, and *Solanum melongena* exhibited a relative insensibility against 3-AT since its activity was decreased only by 19–30%, implying a bifunctional catalase-peroxidase. However, the activity of the root enzyme from *Apium graveolens* and *Petroselinum crispum* was decreased by 85 and 87%, respectively, corresponding to the leaf CAT sensitivity profile.

The results from *Lactuca sativa*, *Cichorium endivia*, *Lycopersicon esculentum*, and *Solanum melongena* are in accordance with recent findings of our laboratory on *Ocimum basilicum* [6]. CAT isoenzymes with enhanced peroxidatic activity and resistance against 3-AT have been also reported from other plants as maize, barley and tobacco but all of them were leaf isoenzymes [7,9,12]. Although a 3-AT resistant CAT has also been detected in young maize roots, it has been reported that as roots mature and grow, the CAT activity decreases and rapidly drops below assay sensitivity [13]. Considering that (a) the expression of the monofunctional CAT is reported to be light-dependent while that of the bifunctional one is not [12] and (b) the bifunctional enzyme is expressed preferentially in dark-grown leaves [13], it is expected that the catalase-peroxidase isoenzyme would predominate in roots. Since catalase-peroxidases have been reported to be located in mitochondria [11] and H_2_O_2_ formation is generated by the mitochondrial respiration [14], the physiological role of these root isoenzymes might be associated with root respiration. Root respiration provides the energy for root growth and for ion absorption and transport and varies depending on the different soil conditions and plant anatomical and biochemical characteristics [15]. Thus, root respiration represents a major source of loss to the atmosphere of the photosynthetically assimilated CO_2_, in some cases even up to 52% [16]. Findings from different plant species that reveal higher root than leaf respiration rate [17] imply a crucial role of catalase-peroxidases on root antioxidant defense, especially under conditions of high respiration rate.

On the other hand, the root CAT sensitivity or insensitivity against 3-AT appears to be similar among species of the same family. Root catalases from Asteraceae and Solanaceae families are insensitive, while the Apiaceae ones are sensitive. These results suggest that the root CAT response against 3-AT is correlated with inherent plant characteristics.

**Figure 2:**
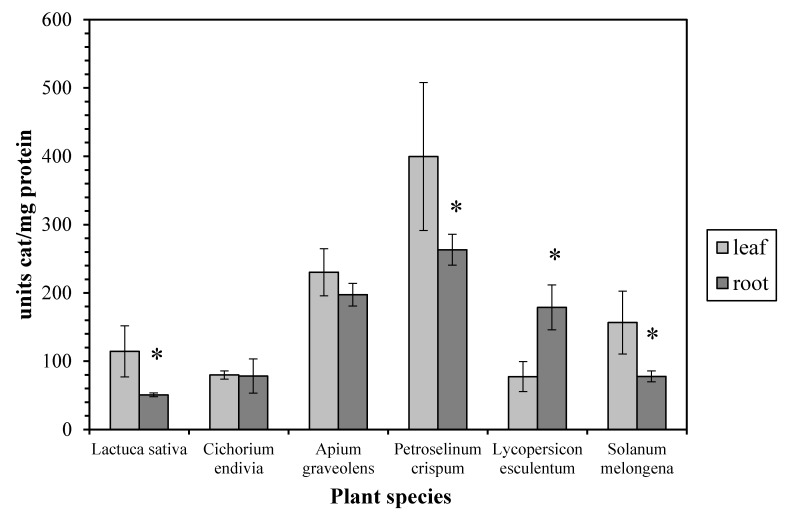
Catalase activity in leaves and roots. Vertical bars represent mean ± SEM (*n* ≥ 3). Means with an asterisk are significantly different from the corresponding leaf activity (*p* < 0.05).

In regard to the enzyme activity profile, we found that it was quite distinct among the examined plant species. Thus, the leaf CAT activity from *Lactuca sativa*, *Petroselinum crispum*, and *Solanum melongena* is higher than the root CAT, while they are similar in *Cichorium endivia* and *Apium graveolens*. In contrast, the leaf CAT activity is lower than the root CAT in *Lycopersicon esculentum* (Figure 2). According to the bibliography, the CAT activity in leaves is usually higher than that in roots as has been reported for maize [4], basil [6], and barley [18]. On the other hand, it seems that, apart from the possible inherent catalase characteristics of each plant species, the plant growth stage may be crucial to the leaf/root CAT activity ratio since it has been reported that (a) the young roots of two different species of the genus *Citrus* display higher CAT activity than the young leaves [19] and (b) in older basil plants the leaf CAT activity is similar to the root one while in younger plants the leaf activity is higher [6].

**Figure 3:**
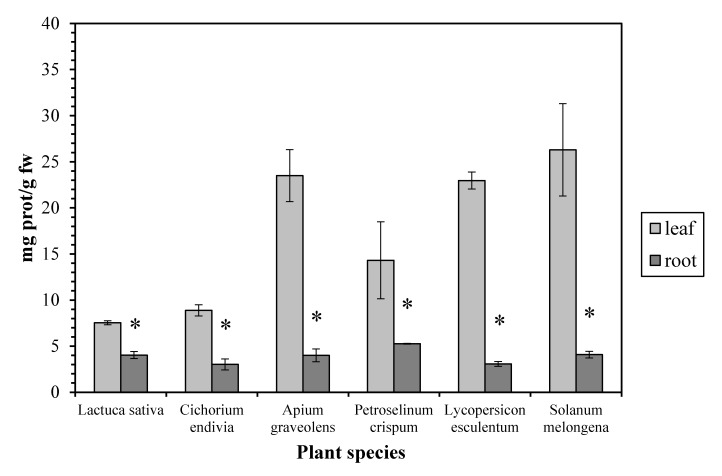
Protein concentration in leaves and roots. Vertical bars represent mean ± SEM (*n* ≥ 3). Means with an asterisk are significantly different from the corresponding leaf concentration (*p* < 0.05).

The protein concentration proved to be higher (up to 7.5-fold) in the leaves than in the roots, in all the examined plant species (Figure 3). Our results are in accordance with measurements in other plants as maize [20], watercress [21], and green bean [22], while similar protein concentrations in leaves and roots have been reported in *Pistia stratiotes*. Proteins (together with membrane lipids) are especially prone to attack by free radicals and are considered as reliable indicators of oxidative stress in plants [23]. The high leaf protein concentration can be explained considering that, although the root is the main site of NO_3_^−^ uptake, considerable data indicate that, for many higher plants, the shoot is the main site of NO_3_^−^ assimilation, as it is the site of amino acid and protein biosynthesis [24].

## 4. Conclusions

In conclusion, the different catalase activity profiles between leaves and roots of several plants may reflect the dominance of different isoenzymes, as it is affirmed by their differential sensitivity against 3-AT. Thereby, it seems that in the roots a bifunctional catalase-peroxidase isoenzyme predominates while leaves are characterized by a typical monofunctional catalase. Considering that catalase is part of the plant antioxidative defense, the above difference between leaf and root isoenzymes possibly corresponds to differences in their physiological role. However, further biochemical and molecular studies about the differential response against 3-AT, not only between leaf and root catalases but also among the root enzymes of different plant families, are needed.
